# PCGF1 Promotes the Tumorigenesis of Malignancies Through Upregulating CCDC34 in Glioma

**DOI:** 10.1002/acn3.70559

**Published:** 2026-10-21

**Authors:** Yuanbing Chen, Jianbing Xiong, Ziran Ou, Tianhao Zhao, Hui Li, Jun Huang, Zhi Tian, Wuyang Cao

**Affiliations:** ^1^ Department of Neurosurgery Third Xiangya Hospital, Central South University Changsha Hunan China; ^2^ Hunan Provincial University Key Laboratory of the Fundamental and Clinical Research on Neurodegenerative Diseases Changsha Medical University Changsha Hunan China; ^3^ Department of Emergency Xiangya Hospital, Central South University Changsha Hunan China; ^4^ National Clinical Research Center of Geriatric Disorders Xiangya Hospital, Central South University Changsha Hunan China; ^5^ Department of Neurosurgery The First Affiliated Hospital of University of South China Hengyang Hunan China; ^6^ Department of Neurosurgery Xiangya Hospital, Central South University Changsha China; ^7^ Department of Neurosurgery First Affiliated Hospital of Jishou University Jishou Hunan China; ^8^ Department of Neurosurgery Changde Hospital, Xiangya School of Medicine, Central South University (The First People's Hospital of Changde City) Changde Hunan China

**Keywords:** CCDC34, glioma, PCGF1, prognosis

## Abstract

**Background:**

Polycomb group factor 1 (PCGF1) and coiled‐coil domain‐containing protein 34 (CCDC34) have been implicated in tumorigenesis in multiple malignancies. However, their biological functions and prognostic significance in glioma remain poorly defined.

**Methods:**

RNA‐sequencing data from glioma cell lines with PCGF1 knockdown were obtained from the GEO database. Correlations between PCGF1 and CCDC34 expression were analyzed using datasets from TCGA, CGGA, and GEO. In addition, RT‐qPCR was performed to quantify PCGF1 and CCDC34 expression in glioma specimens. Kaplan–Meier survival analyses were conducted to evaluate the prognostic value of CCDC34. Functional assays were performed using cell lines with PCGF1 or CCDC34 knockdown and overexpression to assess their roles in glioma progression and to elucidate their regulatory relationship. Cell proliferation and colony formation assays were carried out to evaluate tumorigenic capacity.

**Results:**

CCDC34 expression was significantly downregulated in glioma cell lines following PCGF1 knockdown compared with controls. CCDC34 expression positively correlated with WHO tumor grade in glioma. Elevated CCDC34 expression was significantly associated with poorer patient outcomes. Functionally, CCDC34 expression was closely linked to enhanced cell proliferation and colony‐forming ability. Rescue experiments demonstrated that PCGF1 promotes glioma cell proliferation in a CCDC34‐dependent manner. Gene set enrichment analysis (GSEA) further revealed that both PCGF1 and CCDC34 expression were positively associated with hypoxia, coagulation, and epithelial–mesenchymal transition (EMT) signaling pathways.

**Conclusion:**

Our findings demonstrate that PCGF1 promotes glioma proliferation in a CCDC34‐dependent manner and suggest that CCDC34 may serve as a novel prognostic biomarker and potential therapeutic target in glioma.

## 1 Introduction

Glioma is the most common malignant primary brain tumor and accounts for approximately 80% of all malignant central nervous system tumors [1]. Glioblastoma (GBM) represents the most prevalent and highly aggressive form of glioma in the adult population, with a 5‐year survival rate of only about 5.5%, whereas the 10‐year survival rate can reach up to 90% in patients with low‐grade glioma (LGG) [2, 3]. Thus, according to the WHO grading system, higher‐grade gliomas are generally associated with a poorer prognosis compared to lower‐grade tumors [4]. However, substantial interpatient variability in clinical outcomes persists even among individuals with the same histological grade.

Polycomb group (PcG) proteins act as regulators of gene expression; polycomb group ring finger 1 (PCGF1) is a member of PcG [5, 6, 7]. In previous studies, PCGF1 has been shown to regulate the differentiation and self‐renewal of hematopoietic cells, as well as maintain the stemness of embryonic stem (ES) cells [8, 9, 10]. PCGF1 also plays a role in maintaining stemness in several cancers, such as embryonal carcinoma and oral squamous cell carcinoma [11, 12]. In addition, PCGF1 has been found to promote cancer stem cell self‐renewal and glioma cell proliferation [13, 14], indicating that it could serve as a prognostic biomarker for glioma.

Coiled‐coil domain‐containing protein 34 (CCDC34) contains a coiled‐coil domain that functions in regulating cell movement and signal transduction [15]. Overexpression of CCDC34 promotes the proliferation and metastasis of hepatocellular carcinoma [16]. In colorectal cancer, high CCDC34 expression is correlated with tumor growth, apoptosis, and invasion [17]. Moreover, it is important to note that elevated CCDC34 is associated with poor survival in cervical cancer, pancreatic adenocarcinoma, and esophageal squamous cell carcinoma [18, 19, 20]. However, the expression and function of CCDC34 in glioma have not yet been reported.

Importantly, the relationship between PCGF1 and CCDC34 remains unclear. Thus, the aim of this study was to identify the relationship between PCGF1 and CCDC34, as well as the prognostic value of CCDC34 in glioma, based on previously published databases. Additionally, in vitro assays were performed to elucidate the functional role of CCDC34 in glioma.

## 2 Materials & Methods

### 2.1 Bioinformatics Prediction

To detect the downstream gene of PCGF1, the data of RNA‐seq with the knockdown of PCGF1 in glioma cell lines from the GEO database (GSE121463) would be used. The mRNA expression data of PCGF1 and CCDC34 were downloaded from The Cancer Genome Atlas (TCGA) database, CGGA, and GEO, which would also be used to analyze the correlation between PCGF1 and CCDC34. Overall survival (OS) was defined as the time from diagnosis/surgery to death from any cause or the last follow‐up. Patients who were alive at the last follow‐up were treated as right‐censored observations. Survival curves were estimated using the Kaplan–Meier method and compared using the log‐rank test.

### 2.2 Patients in the Study

A total of 68 patients who had not received any treatment prior to surgery had their tumor tissues removed, and their tissue samples were stored at −80°C. Primary gliomas were surgically removed at Xiangya Hospital between March 2017 and January 2021. Patients were either contacted for follow‐up or attended outpatient visits. Gliomas were classified according to the WHO grading system: grade 2 (*n* = 20), grade 3 (*n* = 16), and grade 4 (*n* = 32) (Table S1). The Research Ethics Committee of Xiangya Hospital approved this study (No. 202112184).

### 2.3 Cell Culture, Plasmids, and shRNAs

The DMEM medium (Gibco, #C1995500BT, Switzerland) supplemented with 10% fetal bovine serum (FBS, Sigma, #12103C, USA) was used to cultivate the glioma cells U251, U87, U373, HS683, and SHG44. These cell lines were kept in a humidified environment with 5% CO_2_ at 37°C.

Lentiviral shRNA clones targeting human CCDC34 and a nonspecific control (GV248) were obtained from GeneChem (Shanghai, China). PCGF1‐ and CCDC34‐overexpressing plasmids were constructed by cloning PCGF1 and CCDC34 cDNA into the pLVX‐EF1α‐IRES‐Puro vector (cat#631988, Clontech). Following the manufacturer's guidelines, lentiviral particles were generated in 293 T cells using Lipofectamine2000. Lentiviral particles were transfected into SHG44, U251, and U87 cells that had been pre‐seeded in six‐well plates in the presence of 10 μg/mL polybrene. Using 2 μg/mL of puromycin, colonies that were consistently expressed were chosen. The sequences of shCCDC34#1: TGAAGATGCCCATGATTCA, shCCDC34#2: CCATGGGAGGTGTGGTTTATT.

### 2.4 RNA Isolation and Quantitative Real‐Time PCR (RT‐qPCR)

With the aid of RNAiso Plus (Takara, #9109, Japan), total RNA was extracted. The PrimeScriptTMRT reagent Kit with gDNA Eraser (Takara, #RR047A, Japan) manufacturer's protocol was followed to isolate the gRNA and reverse transcribe 1 μg of it into cDNA. Using a 7500 Fast Real‐Time PCR System (Applied Biosystems, Life Technologies, USA) and Fast Start Universal SYBR Green Master (Roche, #4913914001, Switzerland), RT‐qPCR was carried out. The relative gene expression levels were normalized to β‐actin. The following primers:

PCGF1 forward sequence: ACGAGACACAGCCACTGCTCAA and reverse sequence: TCCAAACCTCGGGACTGGTAGA.

CCDC34 forward sequence: AAAGTGGCGAGCCTGAGAGGAA and reverse sequence: ATGGTGTCAGGCGGCTTTCTGG.

β‐actin forward sequence: CACCATTGGCAATGAGCGGTTC and reverse sequence: AGGTCTTTGCGGATGTCCACGT.

### 2.5 Western Blot (WB)

Immunoprecipitation (IP) lysis buffer was used to precipitate and lyse the glioma cells. 30 mg of total protein was separated by electrophoresis on 10% SDS‐PAGE gels and then transferred to PVDF (polyvinylidene fluoride). These membranes were incubated with primary antibodies against CCDC34 (Bioss, #bs‐8098R, 1:1000, China), PCGF1 (SANTA CRUZ, #sc‐515,371, 1:1000, USA), and β‐actin (Sigma, #A5441, 1:10000, USA). Subsequently, the membranes were incubated with secondary antibodies for 1 h at room temperature, followed by three washes with PBST. Chemiluminescent signals were determined using the ChemiDox XRS+ imaging system (Bio‐Rad, USA).

### 2.6 Cell Proliferation Assays and Colony Formation Assays

Centrifugation was used to precipitate and count the glioma cells. Five hundred cells per well were seeded onto 96‐well plates using complete medium (200 μL) supplemented with 10% fetal bovine serum. The CellTiter 96 AQueous One Solution Cell Proliferation Assay (Promega, #G3588, USA) was used to measure the vitality of the cells.

Moreover, 500 cells were seeded onto six‐well plates with complete medium. To allow colony formation, the cells were cultured for 14 days at 37°C in a humidified incubator with 5% CO_2_. The colonies were then fixed with methanol for 10 min and stained with crystal violet for 15 min. Colony counting and imaging were performed using ImageJ.

### 2.7 Statistical Analysis

Statistical analyses were performed using GraphPad Prism 8.0 and IBM SPSS Statistics 21.0. Differences between two groups were assessed using the independent‐samples *t*‐test. Correlations between PCGF1 and CCDC34 expression were evaluated using Pearson correlation analysis; simple linear regression lines were fitted for visualization in the scatter plots. The *R* ^2^ values shown in the figures were obtained from the corresponding linear regression models and represent the proportion of variance explained by the fitted model. Survival curves were estimated using the Kaplan–Meier method and compared using the log‐rank test. A two‐sided *p* < 0.05 was considered statistically significant.

## 3 Results

### 3.1 PCGF1 Targets CCDC34 and Upregulates Its Expression in Glioma Cell Lines U87 and U251

To detect the downstream gene of PCGF1, the data of RNA‐seq with the knockdown of PCGF1 in glioma cell lines from the GEO database (GSE121463) were analyzed. After data processing, a total of 687 genes exhibited differential expression between the two groups. A volcano plot was used to visualize these changes, revealing 245 downregulated genes and 422 upregulated genes (Figure 1a). From this analysis, we selected the top 20 genes with significant implications for further analysis (Figure 1a). Four genes that had not been previously studied in glioma were identified. Further validation experiments demonstrated that overexpression of PCGF1 significantly increased the mRNA levels of CCDC34 (Figure S1a; Table S2). To further demonstrate the relationship between PCGF1 and CCDC34, we analyzed the mRNA expression of PCGF1 and CCDC34 in the TCGA, CGGA, and GEO datasets, which revealed a positive correlation between their expression levels (Figure 1b–d). A similar relationship was detected in clinical glioma tissues (Figure 1e). Notably, overexpression of PCGF1 significantly enhanced CCDC34 expression at the level of mRNA and protein in U87 and U251 cells (Figure 1f,g). These results showed that CCDC34 is a downstream target of PCGF1 in glioma.

**FIGURE 1:**
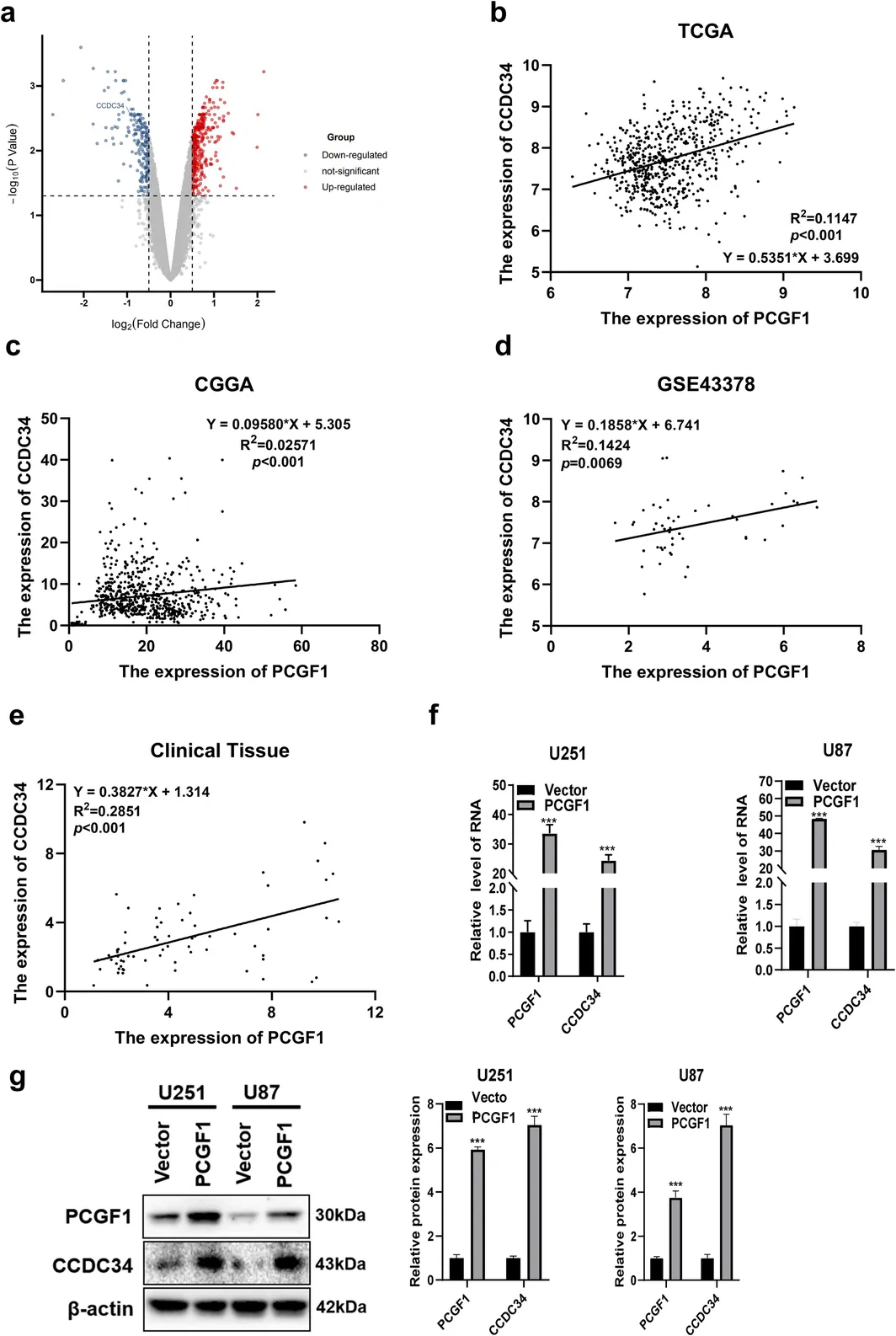
PCGF1 targets CCDC34 and upregulates its expression in glioma cell lines U87 and U251. (a) A volcano plot was used to visualize the downstream gene of PCGF1. (b–e) The relationship between PCGF1 and CCDC34 was analyzed in the TCGA (b), CGGA (c), GEO (d), and clinical tissue samples (e). (f) The RT‐qPCR was used to identify that the overexpression of PCGF1 enhanced the mRNA level of CCDC34 expression in U87 and U251 cells. (g) Western blotting was conducted to determine that the overexpression of PCGF1 enhanced CCDC34 expression in U87 and U251 cells.

### 3.2 The Expression Levels of CCDC34 in Glioma

We analyzed CCDC34 mRNA expression across various histological grades using data from the TCGA, CGGA, and GEO (GSE43378) databases. A positive correlation was observed between tumor grade and CCDC34 expression (Figure 2a–c). Specifically, grade 2 gliomas exhibited lower levels of CCDC34 mRNA expression. To further support the expression of CCDC34 in gliomas, RNA was extracted from tissue samples stored at −80°C. RT‐qPCR analysis revealed that CCDC34 expression was significantly higher in tumors with advanced histological grades (Figure 2d). To further clarify the role of CCDC34 in glioma, WB was used to detect the expression of CCDC34 in 5 pairs of glioma tissues and paracancerous normal tissues. Upregulation of CCDC34 was observed in glioma tissues (Figure 2e). When considered together, the tumor grade and the mRNA expression of CCDC34 showed a positive correlation.

**FIGURE 2:**
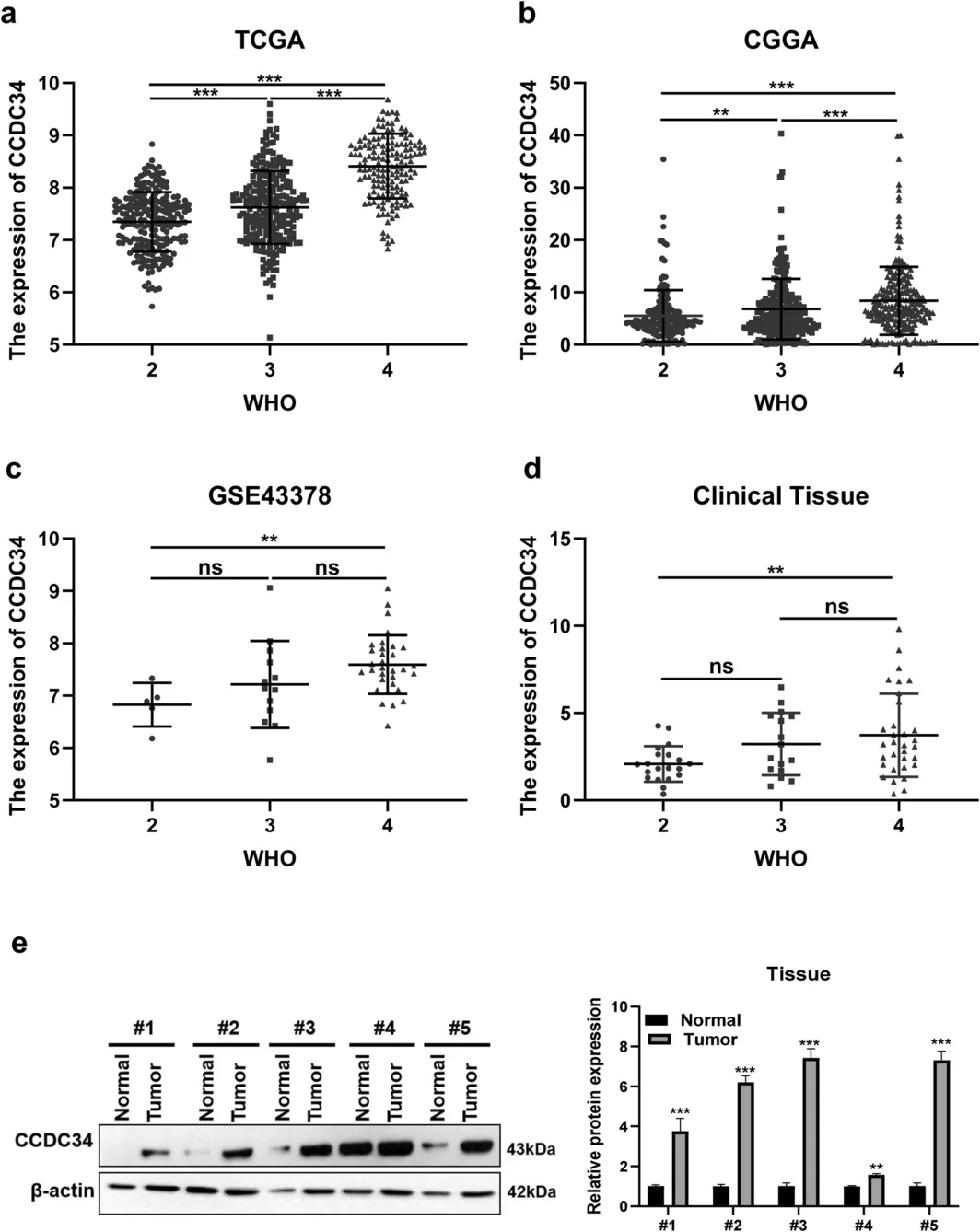
The expression levels of CCDC34 in glioma. The expression of CCDC34 in glioma were detected in CGGA database (a), TCGA database (b), GEO database (c), and clinical patients (d). The protein level of CCDC34 was detected in glioma and non‐tumor brain tissues (e).

### 3.3 High Expression of CCDC34 Associated With Worse Prognosis in Glioma

Kaplan–Meier plots based on the TCGA, CGGA, and GEO (GSE43378) databases were used for the survival study. The findings revealed that low expression of CCDC34 was associated with prolonged survival, in contrast to high expression of CCDC34, which correlated with shorter survival time (Figure 3a–c). The mRNA expression levels of CCDC34 were evaluated in 68 glioma patients with varying tumor grades, who were subsequently followed for survival analysis. Elevated CCDC34 expression consistently correlated with reduced OS. Significant differences in OS were observed between the groups with high and low CCDC34 expression (Figure 3d). Taken together, these results suggest a significant association between CCDC34 expression and patient OS.

**FIGURE 3:**
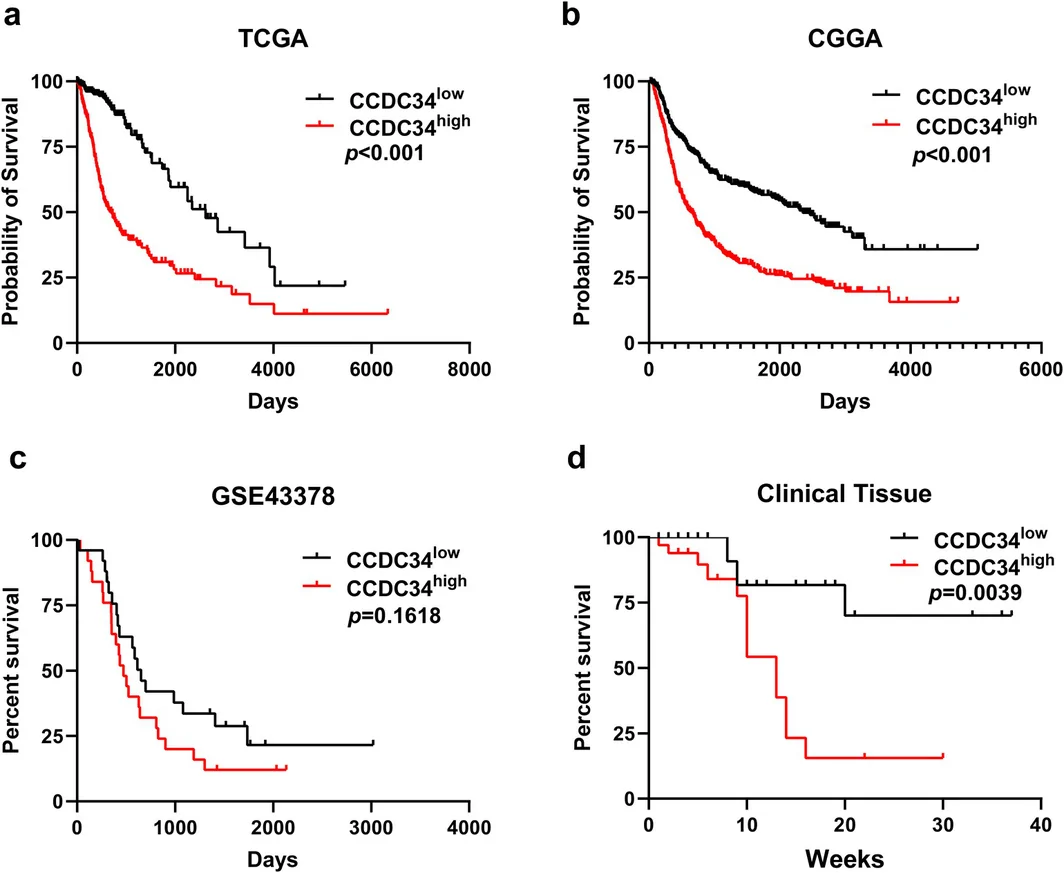
High expression of CCDC34 associated with worse prognosis in glioma. Kaplan–Meier analysis revealed overall survival (OS) curves of glioma patients with different expressions of CCDC34 in CGGA database (a), TCGA database (b), GEO database (c), and clinical patients (d).

Based on data from the TCGA and CGGA databases, stratified analyses of CCDC34 expression and patient prognosis were conducted according to WHO grade. In LGGs (WHO grades II and III), elevated CCDC34 expression was consistently associated with reduced OS (Figure S2a,b and Figure S3a,b). In contrast, no statistically significant association between CCDC34 expression and prognosis was observed in high‐grade GBM (Figure S2c and Figure S3c). These findings suggest that CCDC34 may serve as a potential prognostic biomarker in LGGs. IDH mutation is a key favorable prognostic biomarker in gliomas. Accordingly, further analysis revealed that CCDC34 expression is associated with IDH mutation, and CCDC34 expression is associated with prognosis in both IDH‐mutant and IDH‐wild‐type patients (Figure S2d–f).

To further identify factors influencing survival outcomes in glioma patients, univariate and multivariate Cox regression analyses were performed using data from the TCGA and CGGA cohorts, as well as clinical samples. Multivariate analysis demonstrated that elevated CCDC34 expression was an independent predictor of poor prognosis in glioma patients (*p* < 0.05) (Tables S3–S5).

### 3.4 CCDC34 Expression Is Associated With Proliferative Capacity in Glioma Cells

To elucidate the function of PCGF1 in glioma, U251 and U87 cell lines were stably overexpressed with PCGF1 by using lentivirus, and WB was performed to detect the efficiency of overexpression (Figure 1f). MTS assays were conducted, and the results demonstrated that overexpression of PCGF1 in U251 and U87 cell lines significantly promoted cell proliferation (Figure 4a,b). Additionally, PCGF1 overexpression was found to substantially enhance colony formation in both cell lines (Figure 4c,d).

**FIGURE 4:**
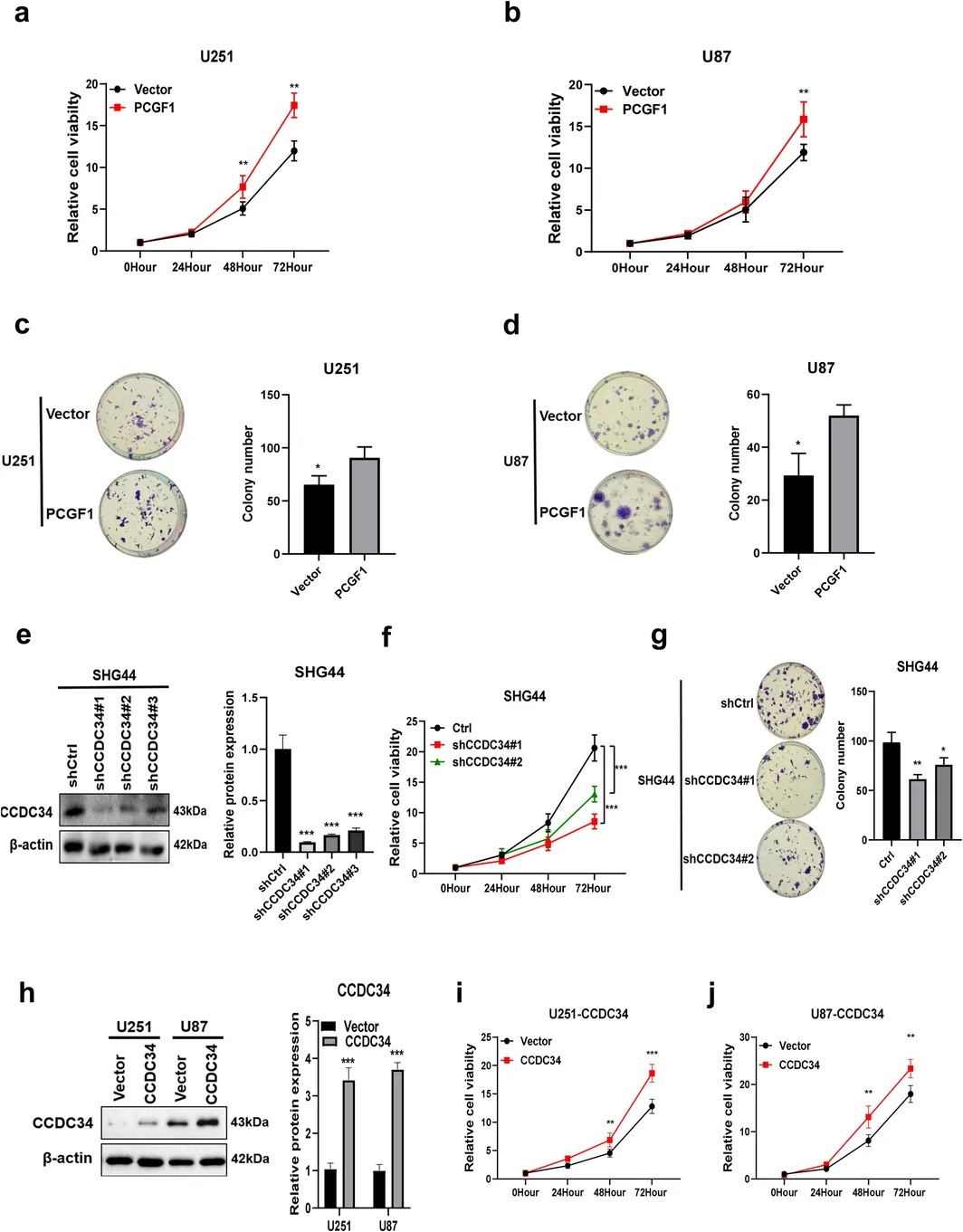
The expression of CCDC34 is associated with the ability of proliferation in glioma cells. (b) The cell viability assay showed that overexpression of PCGF1 promoted cell proliferation in U251 (a) and U87 (b) cells. (c, d) Colony formation assays were performed in U251 and U87 cells stably overexpressing PCGF1. (e) The WB was conducted to determine the level of CCDC34 in SHG44. (f) The cell viability assay showed that knockdown of CCDC34 suppressed cell proliferation in SHG44 cells. (g) Colony formation assays in SHG44 cells with CCDC34 knockdown showed reduced cell proliferation. (h) Western blotting was used to determine CCDC34 levels in U251 and U87 cells. (i, j) The MTS assay demonstrated that overexpression of CCDC34 enhanced proliferative capacity in U251 (i) and U87 (j) cells.

The expression of CCDC34 was identified in glioma cell lines (U251, U87, HS683, SHG44, and U373); we found that SHG44 exhibited the highest expression of CCDC34 (Figure S1b). SHG44 cell lines were stable knockdowns of CCDC34 with two separate sequences by using lentivirus, and WB was performed to detect the efficiency of knockdown (Figure 4e). To assess whether silencing CCDC34 affected glioma cell proliferation, MTS assays were performed. Knockdown of CCDC34 in SHG44 cells significantly inhibited cell growth (Figure 4f). Additionally, downregulation of CCDC34 markedly reduced colony formation in glioma cells (Figure 4g). Additionally, U251 and U87 cell lines stably overexpressing CCDC34 were established, and WB was performed to verify the overexpression efficiency (Figure 4h). Overexpression of CCDC34 in U251 and U87 cell lines was shown to promote cell growth significantly, which was verified by the MTS assays (Figure 4i,j). These results showed that CCDC34 expression affects the proliferative capacity of glioma cell lines.

### 3.5 PCGF1 Promotes Glioma Proliferation in a CCDC34‐Dependent Manner

To further elucidate the relationship between PCGF1 and CCDC34, a rescue experiment was performed. The results demonstrated that PCGF1 upregulated CCDC34 protein levels. However, depletion of CCDC34 in cells stably overexpressing PCGF1 led to decreased CCDC34 expression without significantly affecting PCGF1 expression (Figure 5a). Furthermore, MTS and colony formation assays were performed. We found that PCGF1 promoted glioma proliferation, while depletion of CCDC34 in PCGF1‐overexpressing cells inhibited this effect (Figure 5b,c), suggesting that PCGF1 promotes glioma proliferation in a CCDC34‐dependent manner.

**FIGURE 5:**
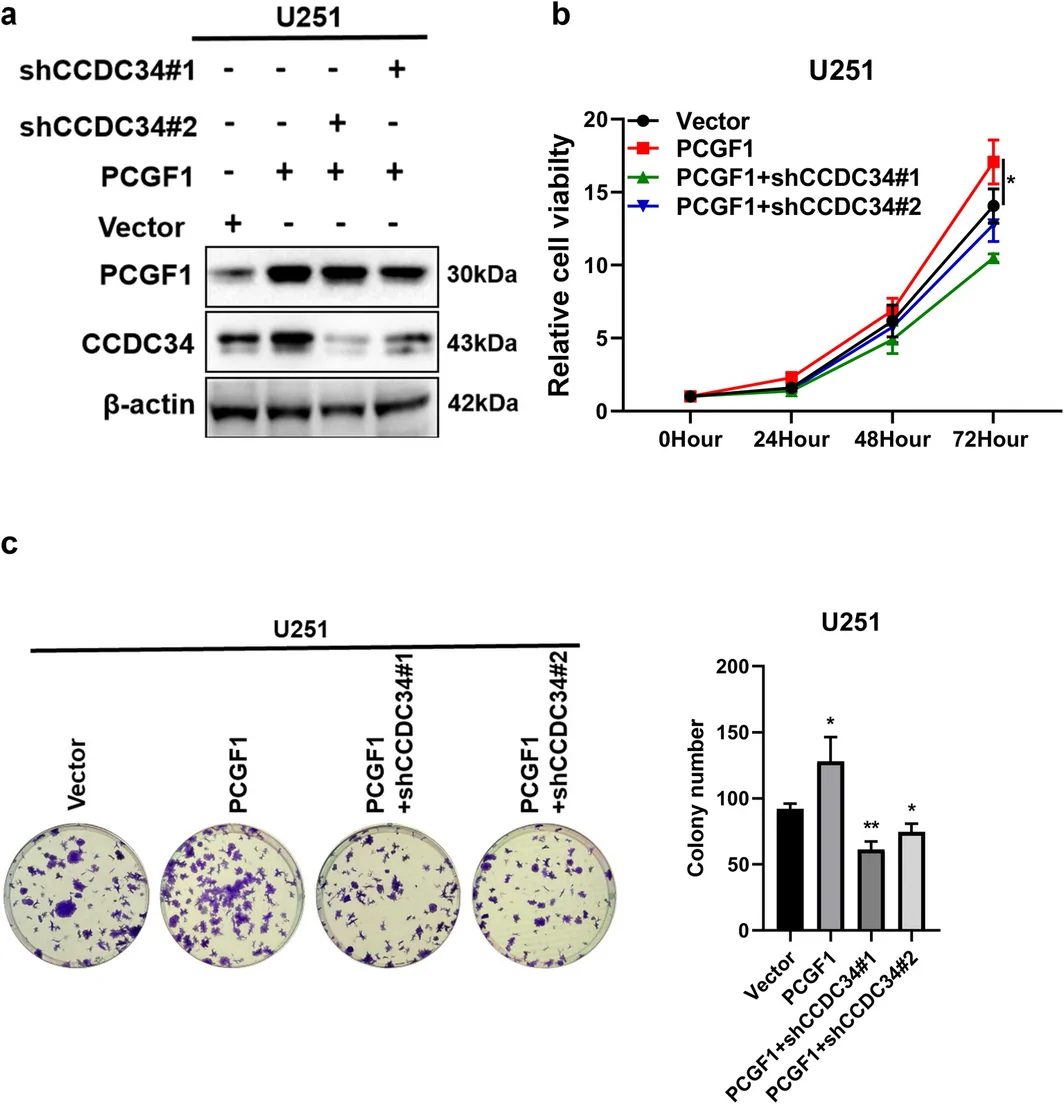
PCGF1 promotes the proliferation of glioma dependent on CCDC34. (a) A rescue experiment was performed. Depletion of CCDC34 in the stably overexpressing PCGF1 cells resulted in downregulation of CCDC34 expression and didn't affect the expression of PCGF1. (b) Cell viability detected by MTS assay in U251. (c) A colony‐formation assay in U251.

### 3.6 Potential Downstream Pathways of PCGF1 and CCDC34

To preliminarily investigate the potential downstream pathways of PCGF1 and CCDC34, the TCGA and CGGA databases were stratified based on high and low expression levels of PCGF1 and CCDC34. Gene Set Enrichment Analysis (GSEA) revealed that PCGF1 and CCDC34 expression in both databases was significantly associated with eight key signaling pathways (Figure 6a). Furthermore, we analyzed RNA‐seq data from PCGF1‐knockdown glioma cell lines in the GEO database (GSE121463), and GSEA suggested that PCGF1 expression was correlated with 10 signaling pathways (Figure 6b–e). Integrative analysis of the three datasets revealed that PCGF1 and CCDC34 expression were positively correlated with hypoxia, coagulation, and epithelial–mesenchymal transition (EMT) signaling pathways (Figure 6b–e).

**FIGURE 6:**
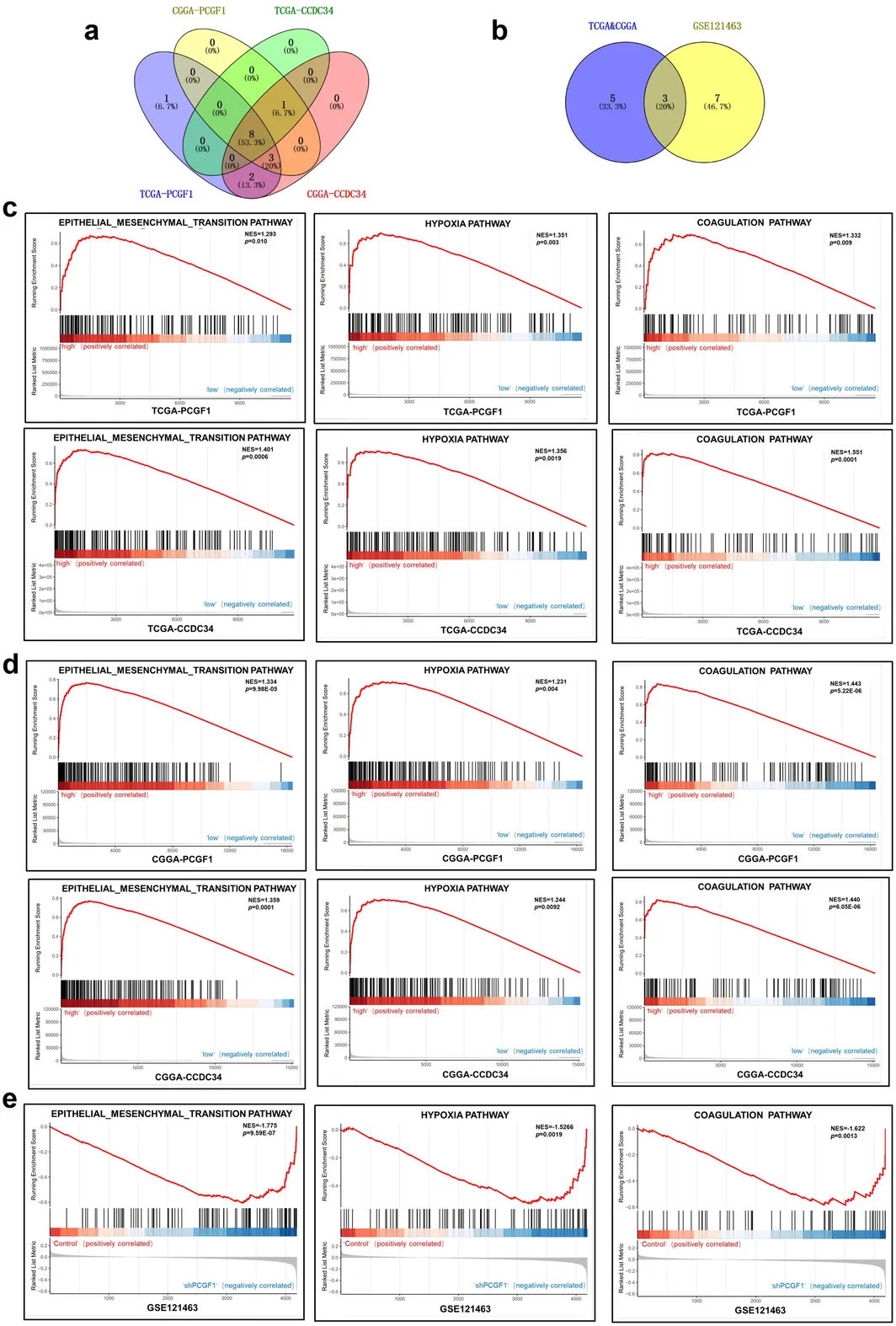
Potential downstream pathways of PCGF1 and CCDC34. (a) GSEA suggests that the expression of PCGF1 and CCDC34 was correlated with 8 pathways in TCGA and CCGA databases. (b) The expression of PCGF1 and CCDC34 was positively correlated with the hypoxia, coagulation, and EMT signaling pathway. (c) The expression of PCGF1 and CCDC34 were positively associated with the pathway of hypoxia, coagulation, and EMT signaling pathway in TCGA. (d) The expression of PCGF1 and CCDC34 were positively associated with the pathway of hypoxia, coagulation, and EMT signaling pathway in CGGA. (e) The downregulated of PCGF1 was positively associated with the pathway of hypoxia, coagulation, and EMT signaling pathway in GSE121463.

## 4 Discussion

According to the WHO pathological grading system, gliomas are classified into four grades (1–4). Grades 1–2 are considered LGG, while grades 3–4 are classified as high‐grade gliomas [21]. In the past decade, numerous studies have focused on identifying new biomarkers and pathways to improve prognostic accuracy and treatment strategies in glioma [22, 23, 24, 25]. However, ideal prognostic markers and underlying molecular mechanisms in glioma remain to be fully elucidated.

With the continuous advancement of technology, the potential of multi‐omics to transform disease treatment and improve patient survival continues to grow, making it a key direction in the current fight against diseases [26]. In this study, we explored the relationship between PCGF1 and CCDC34 through transcriptomics and analyzed the expression of CCDC34 in the TCGA, CGGA, and GEO databases. The expression of CCDC34 was shown to be positively correlated with the histological grades of gliomas. Further, RT‐qPCR analysis showed that patients with higher histological tumor grades exhibited elevated expression of CCDC34. Additionally, rescue experiments demonstrated that PCGF1 promotes glioma proliferation in a CCDC34‐dependent manner. These findings suggest that PCGF1 enhances tumorigenesis in glioma by upregulating CCDC34.

Previous studies have shown that PCGF1 possesses the ability to promote cancer progression [27]. Regarding the potential mechanism of PCGF1, its transcriptional repression activity is one of its most important functions [28]. Furthermore, PCGF1 may interact with other proteins, thereby amplifying its regulatory effects [27]. Interestingly, we found that PCGF1 upregulates CCDC34 rather than functioning solely as a transcriptional repressor. Moreover, overexpression of PCGF1 significantly increased CCDC34 expression at both mRNA and protein levels in glioma cells, suggesting that PCGF1 may also exert transcriptional regulatory effects in glioma. Further experimental validation is needed to confirm this hypothesis.

CCDC34 has been reported to have oncogenic properties in several cancers, such as hepatocellular carcinoma, colorectal cancer, and bladder cancer [29]. The mechanism of CCDC34 in promoting the progression of cancer includes regulating cell movement and cellular signal transduction [15]. However, no previous studies have explored the relationship between CCDC34 and cell proliferation in glioma. In this study, we found that CCDC34 expression was associated with the progression and prognosis of glioma. Furthermore, silencing CCDC34 expression inhibited cell growth and colony formation, while overexpression of CCDC34 significantly promoted cell growth. These results suggest that CCDC34 plays a key role in promoting cell proliferation in glioma.

In this study, GSEA of TCGA, CGGA, and GEO datasets revealed that PCGF1 and CCDC34 expression were positively correlated with hypoxia, coagulation, and EMT signaling pathways. PCGF1 and CCDC34 may affect cancer progression be regulating activation of the EMT process, as demonstrated in a previous studies [16, 30]. Hypoxia is a key feature of the tumor microenvironment and a driving force for metabolic alterations in cancer [31]. The coagulation signaling pathway has been demonstrated to be associated with metastasis and therapy resistance in cancer [32, 33]. Thus, the coagulation and EMT pathways play important roles in cancer development [34, 35]. It was confirmed that CCDC34 functions as an oncogene in glioma, and that PCGF1 promotes glioma proliferation in a CCDC34‐dependent manner. Although no studies have yet revealed the involvement of the hypoxia and coagulation signaling pathways in the regulation by PCGF1 and CCDC34, we hypothesize that PCGF1 may upregulate CCDC34 expression to drive glioma progression by modulating the hypoxia, coagulation, and EMT pathways. However, the exact mechanisms underlying this effect require further investigation. This study has several limitations. First, clinical data were analyzed from samples obtained from 68 patients, which may introduce bias into the statistical results. Second, direct mechanistic evidence demonstrating PCGF1's regulation of CCDC34 in glioma is lacking and requires further investigation. Third, the study did not extensively explore the underlying molecular mechanisms, and there was a lack of in vivo validation, which is essential for definitive confirmation. Therefore, additional studies are needed to further elucidate the regulatory mechanisms, which could potentially be targeted to improve the prognosis of glioma patients.

## Author Contributions

Wuyang Cao, Zhi Tian, and Jun Huang designed the study and wrote the paper. Yuanbing Chen and Jianbing Xiong analyzed the data. Yuanbing Chen collected clinical data and performed follow‐up. Yuanbing Chen and Jianbing Xiong performed in vitro experiments. Hui Li, Ziran Ou, and Tianhao Zhao performed in vivo experiments. All authors contributed to the article and approved the final manuscript.

## Funding

This work was funded by the National Science Foundation of China [82160554(Jun Huang)], Natural Science Foundation of Hunan Province [2025JJ60733(Yuanbing Chen), 2025JJ70620(Zhi Tian)], Major Scientific Research Program for High‐level Talents in Health Sector of Hunan Province [R2023172 (Zhi Tian)], and the Health Commission Fund of Hunan province [202202014633 (Jianbing Xiong), 202304048972 (Wuyang Cao)].

## Ethics Statement

The human material was used in this research, which protected the patient's privacy and met the ethical requirements and was approved by the Research Ethics Committee of the Xiangya hospital (Number: 202112184) and written informed consent was obtained from all subjects. All experiments were conducted according to the relevant guidelines and regulations.

## Consent

The authors have nothing to report.

## Conflicts of Interest

The authors declare no conflicts of interest.

## Supporting information


**Figure S1:** CCDC34 was the downstream gene of PCGF1 in glioma.
(a) RT‐qPCR analysis of downstream target genes in PCGF‐overexpressing U251. (b) WB was used to identify the expression of CCDC34 in different glioma cell lines.
**Figure S2:** High expression of CCDC34 was associated with worse prognosis in glioma in the CGGA cohort.
Kaplan–Meier analysis revealed overall survival (OS) curves of glioma patients with different expressions of CCDC34 in WHO II(a), WHO III(b), and WHO IV(c).
**Figure S3:** High expression of CCDC34 was associated with worse prognosis in glioma in the TCGA cohort.
Kaplan–Meier analysis revealed overall survival (OS) curves of glioma patients with different expressions of CCDC34 in WHO II(a), WHO III(b), and WHO IV(c).
**Table S1:** Baseline information for glioma patients.
Baseline information for glioma patients involved in this study.
**Table S2:** Primers used for RT‐qPCR.
qRT‐PCR primers used for detection of the mRNA level.
**Table S3:** Cox regression analysis of clinicopathological factors affecting the prognosis of patients with glioma in the CGGA cohort.
**Table S4:** Cox regression analysis of clinicopathological factors affecting the prognosis of patients with glioma in the TCGA cohort.
**Table S5:** Cox regression analysis of clinicopathological factors affecting the prognosis of patients with glioma in clinical tissue samples.

## Data Availability

The datasets used analyzed in the present study are included in the manuscript. These data are available in GEO (https://www.ncbi.nlm.nih.gov/geo/), TCGA (https://portal.gdc.cancer.gov/), and CGGA (http://www.cgga.org. cn) databases. The other data analyzed during this study are available from the corresponding author upon reasonable request.
