# Supplementary material for: PCGF1 Promotes the Tumorigenesis of Malignancies Through Upregulating CCDC34 in Glioma
Source: Ann Clin Transl Neurol. 2026 Oct 21:10.1002/acn3.70559. Online ahead of print. doi: 10.1002/acn3.70559 (PMC13687595; doi:10.1002/acn3.70559)
Supplement: Supplementary file 1 [file ACN3-9999-0-s001.docx]

**PCGF1 promotes the tumorigenesis of malignancies through upregulating CCDC34 in glioma**

Yuanbing Chen1,2,#, Jianbing Xiong3,4, #Ziran Ou5, Tianhao Zhao6, Hui Li6, Jun Huang6, Zhi Tian7, Wuyang Cao8*

**Supplemental Figure Legends**

**Figure S1**

**Figure S1. CCDC34 was the downstream gene of PCGF1 in glioma.**

a) RT-qPCR analysis of downstream target genes in PCGF-overexpressing U251. **b)** WB was used to identified the expression of CCDC34 in different glioma cell lines.

**Figure S2**

**Figure S2. High expression of CCDC34 associated with worse prognosis in glioma in CGGA cohort.**

Kaplan–Meier analysis revealed overall survival (OS) curves of glioma patients with different expressions of CCDC34 in WHO II(a), WHO III(b), and WHO IV(c).The expressions of CCDC34 were detected with IDH status in CGGA database (d). Kaplan–Meier analysis revealed overall survival (OS) curves of glioma patients with different expressions of CCDC34 in IDH Wildtype (e) and IDH Mutant (c).

**Figure S3**

**Figure S3. High expression of CCDC34 associated with worse prognosis in glioma in TCGA cohort.**

Kaplan–Meier analysis revealed overall survival (OS) curves of glioma patients with different expressions of CCDC34 in WHO II(a), WHO III(b), and WHO IV(c).

**Supplementary Table S1. Baseline information for glioma patients**

Baseline information for glioma patients involved in this study

| Patient Characters | Patients number |
| --- | --- |
| Total | 68 |
| Age | 47.91 ± 1.564 |
| <50 | 33(48.5%) |
| ≥50 | 35(51.5%) |
| Gender |  |
| Female | 26(38.2%) |
| Male | 42(61.8%) |
| WHO Grade |  |
| II | 20(29.4%) |
| III | 16(23.5%) |
| IV | 32(47.1%) |

**Supplementary Table S2. Primers used for RT-qPCR**

qRT-PCR primers used for detection of the mRNA level

| Gene Name | Sequence |
| --- | --- |
| TPGS2 | F:CCTGATGACCAATGGCTTCCAC |
|  | R:TGGAAGACTGGGTGAGCTGAGT |
| KRTAP1-5 | F:CAGACTAGCTTCTGCGGATTTCC |
|  | R:CAGTTCCGCAGGAGCTGATCTG |
| FTSJ1 | F：TGACACGGGCAGTTGACCTGTG |
|  | R：CACACCTGGTAGTGGAGCCATA |

**Supplementary Table S3. Cox regression analysis of clinicopathological factors affecting the prognosis of patients with glioma in CGGA cohort**

| Clinical characteristics | Univariate analysis | | | Multivariate analysis | | |
| --- | --- | --- | --- | --- | --- | --- |
|  | HR | 95% CI | P | HR | 95% CI | P |
| Age (>50 vs. ≤50) | 0.553 | 0.450-0.680 | ＜0.001 | 0.811 | 0.651-1.009 | 0.060 |
| Gender (Male vs. Female) | 0.939 | 0.768-1.147 | 0.537 | 0.939 | 0.768-1.148 | 0.539 |
| WHO classification | 2.662 | 2.300-3.082 | ＜0.001 | 2.369 | 2.025-2.771 | ＜0.001 |
| CCDC34 expression (High vs. Low) | 0.474 | 0.387-0.581 | ＜0.001 | 0.609 | 0.494-0.752 | ＜0.001 |

**Supplementary Table S4. Cox regression analysis of clinicopathological factors affecting the prognosis of patients with glioma in TCGA cohort**

| Clinical characteristics | Univariate analysis | | | Multivariate analysis | | |
| --- | --- | --- | --- | --- | --- | --- |
|  | HR | 95% CI | P | HR | 95% CI | P |
| Age (>50 vs. ≤50) | 0.237 | 0.182-0.308 | ＜0.001 | 0.456 | 0.340-0.611 | 0.005 |
| Gender (Male vs. Female) | 0.811 | 0.633-1.041 | 0.100 | 0.896 | 0.697-1.151 | 0.389 |
| WHO classification | 0.177 | 0.130-0.240 | ＜0.001 | 0.259 | 0.188-0.358 | ＜0.001 |
| CCDC34 expression (High vs. Low) | 0.393 | 0.303-0.510 | ＜0.001 | 0.678 | 0.517-0.890 | ＜0.001 |

**Supplementary Table S5. Cox regression analysis of clinicopathological factors affecting the prognosis of patients with glioma in clinical tissue**

| Clinical characteristics | Univariate analysis | | | Multivariate analysis | | |
| --- | --- | --- | --- | --- | --- | --- |
|  | HR | 95% CI | P | HR | 95% CI | P |
| Age (>50 vs. ≤50) | 0.396 | 0.141-1.117 | 0.080 | 1.372 | 0329-5.721 | 0.664 |
| Gender (Male vs. Female) | 1.272 | 0.504-3.209 | 0.611 | 1.547 | 0.540-4.433 | 0.417 |
| WHO classification | 0.247 | 0.056-1.085 | 0.064 | 0.235 | 0.047-1.177 | 0.078 |
| CCDC34 expression (High vs. Low) | 0.200 | 0.070-0.570 | 0.003 | 0.219 | 0.051-0.932 | 0.040 |
